# Supplementary material for: Development of limb bone laminarity in the homing pigeon (Columba livia)
Source: PeerJ. 2020 Sep 8;8:e9878. doi: 10.7717/peerj.9878 (PMC7485507; doi:10.7717/peerj.9878)
Supplement: Supplemental Information 1 [file peerj-08-9878-s001.docx]

**Table S1:**

**Age and body mass of analyzed specimens.**

| **Specimen** | **Postnatal age (wk)** | **Body mass (g)** |
| --- | --- | --- |
| MWU 263 | 0 | 15 |
| MWU 261 | 1-2 | 83 |
| MWU 260 | 1-2 | 131 |
| MWU 258 | 2-3 | 242 |
| MWU 267 | 2-3 | 263 |
| MWU 270 | 3-4 | 209 |
| MWU 271 | 3-4 | 209 |
| MWU 272 | 3-4 | 341 |
| MWU 269 | 3-4 | 372 |
| MWU 273 | 4-5 | 314 |
| MWU 276 | 4-5 | 360 |
| MWU 275 | 4-5 | 364 |
| MWU 274 | 4-5 | 374 |
| MWU 256 | 5-6 | 455 |
| MWU 257 | 5-6 | 498 |
| MWU 254 | 8-10 | 482 |
| MWU 255 | 8-10 | 563 |
