# Supplementary material for: Development of limb bone laminarity in the homing pigeon (Columba livia)
Source: PeerJ. 2020 Sep 8;8:e9878. doi: 10.7717/peerj.9878 (PMC7485507; doi:10.7717/peerj.9878)
Supplement: Supplemental Information 2 [file peerj-08-9878-s002.docx]

**Table S2:**

**Cross-sectional and microstructural properties of humeri.**

| **Specimen** | **Circum. (mm)** | **Length (mm)** | **Z_p_ (mm^3^)** | **I_max_/I_min_** | **Porosity (%)** | **LI** |
| --- | --- | --- | --- | --- | --- | --- |
| MWU 263 | 2.934 | 10.9 | 0.059 | 1.800 | 30.1 | n/a |
| MWU 261 | 6.233 | 18.0 | 0.429 | 1.486 | 58.8 | n/a |
| MWU 260 | 7.873 | 22.4 | 0.515 | 1.452 | 74.2 | n/a |
| MWU 258 | 12.330 | 38.3 | 4.058 | 1.699 | 29.1 | 0.348 |
| MWU 267 | 12.201 | 35.4 | 2.905 | 1.659 | 49.5 | n/a |
| MWU 270 | 13.191 | 39.9 | 4.576 | 1.628 | 6.2 | 0.378 |
| MWU 271 | 12.892 | 41.1 | 5.341 | 1.602 | 7.1 | 0.486 |
| MWU 272 | 15.732 | 47.4 | 10.893 | 1.592 | 6.7 | 0.202 |
| MWU 269 | 16.732 | 47.8 | 12.124 | 1.576 | 11.7 | 0.082 |
| MWU 273 | 16.122 | 47.5 | 11.293 | 1.621 | 6.7 | 0.189 |
| MWU 276 | 16.507 | 46.7 | 12.818 | 1.474 | 4.6 | 0.126 |
| MWU 275 | 16.888 | 47.6 | 13.649 | 1.577 | 5.7 | 0.159 |
| MWU 274 | 17.818 | 49.9 | 13.123 | 1.686 | 4.3 | 0.182 |
| MWU 256 | 17.097 | 49.2 | 11.510 | 1.645 | 2.7 | 0.124 |
| MWU 257 | 18.475 | 50.9 | 14.294 | 1.661 | 3.1 | 0.140 |
| MWU 254 | 18.932 | 50.0 | 16.442 | 1.673 | 3.6 | 0.158 |
| MWU 255 | 20.543 | 51.0 | 17.822 | 1.684 | 3.2 | 0.122 |
