# Supplementary material for: Development of limb bone laminarity in the homing pigeon (Columba livia)
Source: PeerJ. 2020 Sep 8;8:e9878. doi: 10.7717/peerj.9878 (PMC7485507; doi:10.7717/peerj.9878)
Supplement: Supplemental Information 3 [file peerj-08-9878-s003.docx]

**Table S3:**

**Cross-sectional and microstructural properties of ulnae.**

| **Specimen** | **Circum. (mm)** | **Length (mm)** | **Z_p_ (mm^3^)** | **I_max_/I_min_** | **Porosity (%)** | **LI** |
| --- | --- | --- | --- | --- | --- | --- |
| MWU 263 | 2.336 | 11.4 | 0.028 | 1.200 | 36.2 | n/a |
| MWU 261 | 4.052 | 19.5 | 0.136 | 1.150 | 54.1 | n/a |
| MWU 260 | 5.706 | 24.3 | 0.230 | 1.109 | 66.0 | n/a |
| MWU 258 | 9.949 | 42.8 | 2.348 | 1.343 | 21.1 | 0.311 |
| MWU 267 | 8.548 | 40.1 | 1.248 | 1.319 | 48.8 | n/a |
| MWU 270 | 10.518 | 45.4 | 2.933 | 1.467 | 5.2 | 0.281 |
| MWU 271 | 10.845 | 46.2 | 3.518 | 1.639 | 6.2 | 0.332 |
| MWU 272 | 12.369 | 54.1 | 5.340 | 1.571 | 6.4 | 0.142 |
| MWU 269 | 13.150 | 54.5 | 6.496 | 1.212 | 10.1 | 0.075 |
| MWU 273 | 12.348 | 54.5 | 5.758 | 1.342 | 6.6 | 0.143 |
| MWU 276 | 12.777 | 54.1 | 6.090 | 1.402 | 3.9 | 0.088 |
| MWU 275 | 13.678 | 54.9 | 7.029 | 1.490 | 5.2 | 0.130 |
| MWU 274 | 12.678 | 56.9 | 5.649 | 1.197 | 3.8 | 0.094 |
| MWU 256 | 13.875 | 56.4 | 7.016 | 1.371 | 3.4 | 0.086 |
| MWU 257 | 14.582 | 56.3 | 7.058 | 1.486 | 4.8 | 0.106 |
| MWU 254 | 15.071 | 58.0 | 7.797 | 1.478 | 2.5 | 0.110 |
| MWU 255 | 14.770 | 56.2 | 8.053 | 1.446 | 2.8 | 0.103 |
