# Supplementary material for: Development of limb bone laminarity in the homing pigeon (Columba livia)
Source: PeerJ. 2020 Sep 8;8:e9878. doi: 10.7717/peerj.9878 (PMC7485507; doi:10.7717/peerj.9878)
Supplement: Supplemental Information 4 [file peerj-08-9878-s004.docx]

**Table S4:**

**Cross-sectional and microstructural properties of radii.**

| **Specimen** | **Circum. (mm)** | **Length (mm)** | **Z_p_ (mm^3^)** | **I_max_/I_min_** | **Porosity (%)** | **LI** |
| --- | --- | --- | --- | --- | --- | --- |
| MWU 263 | 1.541 | 10.0 | 0.008 | 1.117 | 13.8 | 0.400 |
| MWU 261 | 2.385 | 19.4 | 0.032 | 1.600 | 37.6 | n/a |
| MWU 260 | 2.982 | 22.5 | 0.050 | 1.167 | 50.4 | n/a |
| MWU 258 | 5.462 | 40.3 | 0.546 | 1.103 | 13.6 | 0.108 |
| MWU 267 | 4.793 | 36.1 | 0.276 | 1.204 | 38.9 | n/a |
| MWU 270 | 6.239 | 41.1 | 0.734 | 1.266 | 4.6 | 0.088 |
| MWU 271 | 6.433 | 42.4 | 0.878 | 1.153 | 3.7 | 0.129 |
| MWU 272 | 6.823 | 49.0 | 1.281 | 1.130 | 4.8 | 0.067 |
| MWU 269 | 7.427 | 50.3 | 1.501 | 1.345 | 7.8 | 0.039 |
| MWU 273 | 7.062 | 49.5 | 1.380 | 1.323 | 5.4 | 0.087 |
| MWU 276 | 7.807 | 48.3 | 1.709 | 1.316 | 4.1 | 0.029 |
| MWU 275 | 8.310 | 53.4 | 2.158 | 1.265 | 4.7 | 0.034 |
| MWU 274 | 7.675 | 50.6 | 1.481 | 1.379 | 3.8 | 0.079 |
| MWU 256 | 8.513 | 55.3 | 1.845 | 1.202 | 2.5 | 0.024 |
| MWU 257 | 8.912 | 52.4 | 2.205 | 1.372 | 3.0 | 0.063 |
| MWU 254 | 9.034 | 54.0 | 2.266 | 1.053 | 1.9 | 0.029 |
| MWU 255 | 9.206 | 52.3 | 2.516 | 1.335 | 2.3 | 0.096 |
