# Supplementary material for: Development of limb bone laminarity in the homing pigeon (Columba livia)
Source: PeerJ. 2020 Sep 8;8:e9878. doi: 10.7717/peerj.9878 (PMC7485507; doi:10.7717/peerj.9878)
Supplement: Supplemental Information 5 [file peerj-08-9878-s005.docx]

**Table S5:**

**Cross-sectional and microstructural properties of femora.**

| **Specimen** | **Circum. (mm)** | **Length (mm)** | **Z_p_ (mm^3^)** | **I_max_/I_min_** | **Porosity (%)** | **LI** |
| --- | --- | --- | --- | --- | --- | --- |
| MWU 263 | 2.807 | 14.0 | 0.062 | 1.308 | 30.5 | n/a |
| MWU 261 | 2.965 | 25.4 | 0.065 | 1.133 | 32.5 | n/a |
| MWU 260 | 7.524 | 26.5 | 0.694 | 1.151 | 54.9 | n/a |
| MWU 258 | 9.877 | 35.2 | 2.072 | 1.123 | 18.2 | 0.240 |
| MWU 267 | 9.459 | 36.8 | 2.376 | 1.086 | 33.7 | n/a |
| MWU 270 | 9.818 | 35.7 | 2.139 | 1.040 | 4.0 | 0.321 |
| MWU 271 | 9.932 | 35.4 | 2.294 | 1.058 | 4.9 | 0.299 |
| MWU 272 | 11.419 | 42.5 | 4.904 | 1.054 | 4.6 | 0.120 |
| MWU 269 | 11.835 | 42.4 | 4.783 | 1.066 | 6.2 | 0.124 |
| MWU 273 | 11.426 | 44.1 | 4.334 | 1.119 | 4.8 | 0.132 |
| MWU 276 | 11.966 | 41.7 | 5.265 | 1.212 | 3.3 | 0.197 |
| MWU 275 | 12.036 | 41.5 | 5.162 | 1.117 | 3.9 | 0.160 |
| MWU 274 | 12.430 | 44.3 | 5.282 | 1.126 | 2.7 | 0.199 |
| MWU 256 | 12.451 | 44.4 | 5.544 | 1.079 | 2.1 | 0.121 |
| MWU 257 | 12.501 | 45.0 | 6.196 | 1.028 | 3.0 | 0.111 |
| MWU 254 | 13.462 | 45.0 | 7.558 | 1.089 | 2.1 | 0.064 |
| MWU 255 | 13.237 | 45.8 | 7.668 | 1.185 | 2.3 | 0.063 |
