# Supplementary material for: Development of limb bone laminarity in the homing pigeon (Columba livia)
Source: PeerJ. 2020 Sep 8;8:e9878. doi: 10.7717/peerj.9878 (PMC7485507; doi:10.7717/peerj.9878)
Supplement: Supplemental Information 6 [file peerj-08-9878-s006.docx]

**Table S6:**

**Cross-sectional and microstructural properties of tibiotarsii.**

| **Specimen** | **Circum. (mm)** | **Length (mm)** | **Z_p_ (mm^3^)** | **I_max/_I_min_** | **Porosity (%)** | **LI** |
| --- | --- | --- | --- | --- | --- | --- |
| MWU 263 | 3.001 | 15.2 | 0.060 | *1.162 | 38.8.2 | n/a |
| MWU 261 | 4.774 | 28.7 | 0.275 | 1.103 | 41.7 | n/a |
| MWU 260 | 6.223 | 29.8 | 0.466 | 1.217 | 51.0 | n/a |
| MWU 258 | 9.489 | 46.6 | 1.804 | 1.146 | 13.4 | 0.106 |
| MWU 267 | 8.382 | 44.2 | 1.583 | 1.265 | 34.4 | n/a |
| MWU 270 | 8.875 | 48.4 | 1.713 | 1.231 | 3.3 | 0.135 |
| MWU 271 | 9.193 | 47.6 | 1.992 | 1.315 | 3.2 | 0.084 |
| MWU 272 | 10.225 | 59.5 | 3.222 | 1.649 | 8.6 | 0.034 |
| MWU 269 | 11.402 | 59.9 | 4.023 | 1.277 | 4.9 | 0.037 |
| MWU 273 | 9.934 | 59.3 | 3.261 | 1.451 | 4.3 | 0.077 |
| MWU 276 | 11.314 | 59.7 | 4.225 | 1.444 | 2.8 | 0.033 |
| MWU 275 | 12.887 | 64.3 | 5.311 | 1.426 | 9.0 | 0.061 |
| MWU 274 | 10.660 | 61.2 | 3.545 | 1.478 | 2.3 | 0.071 |
| MWU 256 | 11.774 | 60.5 | 4.095 | 1.596 | 7.2 | 0.033 |
| MWU 257 | 11.932 | 62.7 | 4.759 | 1.584 | 2.4 | 0.062 |
| MWU 254 | 11.925 | 61.8 | 5.681 | 1.349 | 5.1 | 0.052 |
| MWU 255 | 12.866 | 63.6 | 6.444 | 1.435 | 3.9 | 0.042 |
